# The Band Effect—Physically Strenuous Music Making Increases Esthetic Appreciation of Music

**DOI:** 10.3389/fnins.2016.00448

**Published:** 2016-10-17

**Authors:** Thomas H. Fritz, Lydia Schneider, Arno Villringer

**Affiliations:** ^1^Max Planck Institute for Human Cognitive and Brain ScienceLeipzig, Germany; ^2^Institute for Psychoacoustics and Electronic Music (IPEM), University of GhentGhent, Belgium; ^3^Department of Nuclear Medicine, University of LeipzigLeipzig, Germany

**Keywords:** positivity bias, musical agency, esthetics, exercise, appreciation

## Abstract

The esthetic appreciation of music is strongly influenced by cultural background and personal taste. One would expect that this would complicate the utilizability of musical feedback in paradigms, such that music would only be perceived as a reward if it complies to personal esthetic appreciation. Here we report data where we assessed esthetic appreciation of music after 1. a physically strenuous music improvisation and 2. after passive music listening (where participants esthetically assessed similar music). Data are reported from two experiments with different patient groups: 1. Drug abuse patients, and 2. Chronic pain patients. Participants in both experiments performed *Jymmin*, a music feedback method where exercise equipment is modified in such a way that it can be played like musical instruments by modulating musical parameters in a composition software. This combines physical exertion with musical performance in a fashion that has previously been shown to have a number of positive psychological effects such as enhanced mood and reduced perceived exertion. In both experiments esthetic appreciation of musical presentations during *Jymmin* and a control condition without musical agency were compared. Data show that both patient groups perceived the musical outcome of their own performance as more esthetically pleasing than similar music they listened to passively. This suggests that the act of making music (when combined with physical exertion) is associated with a positivity bias about the perceived esthetical quality of the musical outcome. The outcome of personal musical agency thus tends to be perceived as rewarding even if it does not comply with personal esthetic appreciation. This suggests that musical feedback interventions may not always have to be highly individualized because individual taste may not always be crucial. The results also suggest that the method applied here may be efficient at encouraging music listeners to actively explore new musical styles that they might otherwise be reluctant to listen to (e.g., avant-garde music). The results also hint toward a deeper understanding of why musicians, who exert themselves physically during musical performances to generate music and regardless of the type of music they are playing, typically find the physically demanding experience esthetically satisfying.

## Introduction

### Influence on musical appreciation

Musical appreciation can be influenced both by the acoustical structure of the musical signal and exposure to human culture. Listeners with a Western cultural background and from a society naïve to Western music have in their esthetic assessment been shown to respond sensitively to variations of acoustical properties of music. A systematic reduction of esthetic appreciation has been shown to be achieved by both a manipulation of the horizontal organization of the music by playing it backward (Fritz et al., 2009, 2014) or scrambling (Menon and Levitin, 2005), and by a manipulation of the vertical organization of the music by increasing sensory dissonance (Koelsch et al., 2006; Fritz et al., 2009, 2013c; Mueller et al., 2011, 2015).

However, it is also apparent that musical appreciation is sensitive to previous exposure to human culture. For example, music listeners tend to have a greater appreciation for music that they are familiar with. This probably also relates to the degree to which they understand musical meaning in a musical excerpt, such that for example Mafa listeners who had never before listened to Western music showed a greater appreciation for the Western music if they were able to identify emotional expressions such as happy, sad, and scary in Western music (Fritz et al., 2009). Note however, that musical meaning related to emotional expressions may universally be better understood compared other types of extra-musical associations that vary more strongly between cultures (Fritz et al., 2013d).

It is interesting to note that creating music by interaction in an improvisation also constitutes a semiotic process where musical meaning is created on the fly such that for example certain combinations of musical gestures refer to a previous situation in the improvisation where they have occurred already (and thus begin to signify abstract aspects of these previous time points). Accordingly, the process of music making is likely to result in a greater richness of semantic association with a musical piece (for example also with respect to the knowledge about how the music was performed on the instruments etc.), and will thus probably rather lead to an enhanced esthetic appreciation. The act of music making may thus also constitute a strong basis for music as a universal means of communication.

### Music as a reward

A number of studies indicate that music is efficient as a reward and neuro-chemically triggers parts of our reward system (Menon and Levitin, 2005; Koelsch et al., 2006; Salimpoor et al., 2011; Mueller et al., 2015). It is therefore reasonable to assume that music will be an efficient reinforcer in the acquisition of new skills and attitudes, e.g., during therapeutic interventions.

### Music feedback paradigms

Musical feedback is a category of acoustic feedback that specifically aims at engaging the participant emotionally during an intervention. Given that there are a number of ways that emotions can be evoked musically (Juslin and Västfjäll, 2008), there is accordingly also a variety of methods by which musical feedback can be emotionally engaging to a performer. This includes for example playing highly familiar melodies on drum pads as a measure to improve motor skills after stroke (Schneider et al., 2007).

Many interventions aimed at engaging individuals emotionally by music feedback and also by music making, however, have to be tailored specifically to each participant, taking into account musical education, motor, and rhythmical skills, and musical taste.

In recent years a musical feedback intervention has been developed further that has been demonstrated to be highly emotionally engaging across individuals and does not require any type of previous musical education and even rhythmical skills, and can quite easily be adapted to individual motor skills (e.g., after stroke or in elder participants). This intervention, called *Jymmin* (mixture of jammin and gym), allows participants to express themselves musically by exercising on fitness machines that have been modified to transform physical movements into musical sounds and thus allow music to be played interactively in a group (Fritz et al., 2013a,b, 2015). Musical expression during this procedure may for example be realized by mapping the degree of weight displacement (thus an alteration of position) onto sound effects to change the timbre of the sound, the pitch of the sound, the number of delays of a sound, etc.

The combination of musical expression and extreme physiological arousal has been shown to create an intense musical and emotional experience that correlated with a decreased sense of exertion (Fritz et al., 2013b). It also created an increased motor effectivity, which could be observed when comparing this condition to a control condition where music was perceived passively during workout (similar to conventional training in fitness studios, Fritz et al., 2013b). *Jymmin* has also been reported to evoke an enhanced mood, a process probably at least partly hormonally mediated (Fritz et al., 2013a). This is comparable to the notion of a runner's high, but is already observed after 10 min of training and does not require training in the weeks preceding the intervention.

Such enhanced mood could also be observed in a patient group listening passively to a recording of a previous *Jymmin* session, more specifically we observed a positive correlation between participants' mood and their desire to engage in social activities with their former training partners after listening to the self-made music (Fritz et al., 2015). Furthermore, listening to the recording increased self-efficacy, and a readiness to engage socially (note, however, that these effects depended on the context in which the recordings were presented).

## Methods

### Experiment 1

#### Participants

Twenty-seven participants (23 male, 4 female) took part in the experiment. Participants' age ranged from 20 to 47 years (*M* = 31.33 years). Clinical archive data showed that 77.7% of the participants used more than two drugs regularly during the pre-clinic period (which is considered polydrug use according to the ICD-10 diagnosis system, number F19.2).

Furthermore, 63% of patients were involved in criminal activities and incarcerated for a time range between 1 and 96 months, and most were doing the rehabilitation program during their prison sentence (§35 in the German Controlled Substances Act). 48.1% of the participants were diagnosed with Attention Deficit Hyperactivity Disorder (ADHD) or a related hyperkinetic as comorbidity.

Informed written consent was obtained from all of the subjects and the experiment was conducted in accordance with the Declaration of Helsinki's ethical principles for research involving humans. It conformed to internationally accepted policy statements regarding the use of human subjects and was approved by the ethics committee of the University of Leipzig.

### Experiment 2

#### Participants

Twenty-four participants (20 female and 4 men; age range 34–64, *M* = 51.67, *SD* = 6.84) took part in the experiment. None of the participants were professional body builders, musicians, or athletes. Clinical data showed that all participants were suffering from chronic pain, which has been defined as pain that persists or recurs for more than 3 months, with no clear physical causes. A co-morbid major depressive syndrome was present in 45.8% of patients (assessed with the *Brief Patient Health Questionnaire*). On the day of the experiment patients' pain levels ranged from 0.3 to 9.4 (*M* = 5.14, *SD* = 3.04) as indicated on a Visual Analog Scale of 0–100 mm. 30.4% of participants suffered from mild pain, 26.1% from moderate pain, and 43.5% from severe pain.

The study adhered to the guidelines of the Declaration of Helsinki and was approved by the ethics committee of the University of Leipzig, Germany. In addition, informed written consent was obtained from all participants.

### Experiment 1 and 2

#### Experimental design

The experiment included two conditions: In one condition the participants worked out on fitness machines while passively listening to music (*non-musical-agency condition*); in a second condition they worked out on fitness machines while listening to a musical feedback of their own movements (*Jymmin condition*). All participants performed both conditions of the experiment, the *Jymmin condition* and the *non-musical-agency condition*. The physical workout was conducted with three different fitness machines, a tower (lat pulldown), an abdominal trainer, and a stepper. All three machines are standard fitness machines that are commercially available and they allow for guided movements.

In the *Jymmin* condition the movements of participants on the fitness machines were mapped to a music composition software (Ableton Live 8) so that the deflection of the fitness machines was translated into musical parameters of an acoustic feedback signal (for a detailed description see Fritz et al., 2013a,b; for sound examples see Supplementary Material). Each fitness machine produced a different musical soundscape, and the combined musical feedback of all three fitness machines created sounds at a constant tempo of 130 bpm (beats per minute) and could interactively be combined into a holistic musical piece. The musical interaction was predefined in terms of sounds to be modulated, the musical parameters to be modulated, and the metric of the music. The music style used in the experiment was rather minimalistic electronic music. While exercising, physical movements of all three patients were transformed by the software into immediate musical feedback depending on the degree of weight displacement. Musical parameters such as pitch and cutoff-filter of the musical sounds played could be altered by the movements (for more detail see Fritz et al., 2013a,b, 2015). Music during the experimental and control condition was audible over loudspeakers.

The musical performances of all patient groups in the *Jymmin* condition were recorded and played back during the *non-musical agency* condition (with exception of the first group who listened to a recording of their own *Jymmin* condition) to ensure a comparable exposure to music during both experimental conditions. That is, participants who had a non-agency condition first heard music from one of the other groups in this condition. The first group that had Jymmin first, listened to their own recorded Jymmin music session in the non-agency condition. Furthermore, we controlled for the sequence in which both conditions were performed, so that half of the patients first performed the *Jymmin* condition, and the other half first performed the *non-musical-agency* condition (cross-over design).

### Experimental procedure

The experimental procedure in both experiments was similar with the exception of questions included in the post-intervention questionnaire that were specific to the patients' deficits. Participants were randomly assigned to different timeslots, such that groups (each consisted of three participants) were formed and tested on two consecutive days. Before participants started with the workout conditions, they were asked to fill out general information items on gender and age. Then participants entered the training room and were asked to choose their preferred fitness machine. A short explanation on how to use the fitness machines correctly in terms of physiologically healthy movements was given by the experimenter, followed by the task instruction: “Use the fitness machines now in a way in which you are physically comfortable.” Each of the conditions was performed for 10 min, during which all participants could see each other and (during the *Jymmin* condition) could hear their own sound and the sound of the other two performers through a speaker system. After each condition patients took a rest and then their esthetical appreciation for the music was assessed with the following question: “how much did you like the music during workout?.” In Experiment 1 the drug abuse patients indicated their esthetic appreciation on a 5-point Likert scale from 1 (“not at all”) to 5 (“very strongly”). In Experiment 2 the chronic pain patients assessed their esthetic appreciation of the music on a Visual Analogue Scale ranging from 1 (“not at all”) to 100 mm (“very strongly”).

### Data analysis

The behavioral data of both experiments were separately analyzed with non-parametric tests using SPSS 22 (IBM). Participants with a missing response were indicated in SPSS and excluded from the corresponding analysis. The data should best be regarded as ordinally scaled, and not interval scaled, we therefore used non-parametric tests. A Sign test was calculated, because the distribution of differences between paired observations was neither normal nor symmetrical.

## Results

### Experiment 1

**Figure 1:**
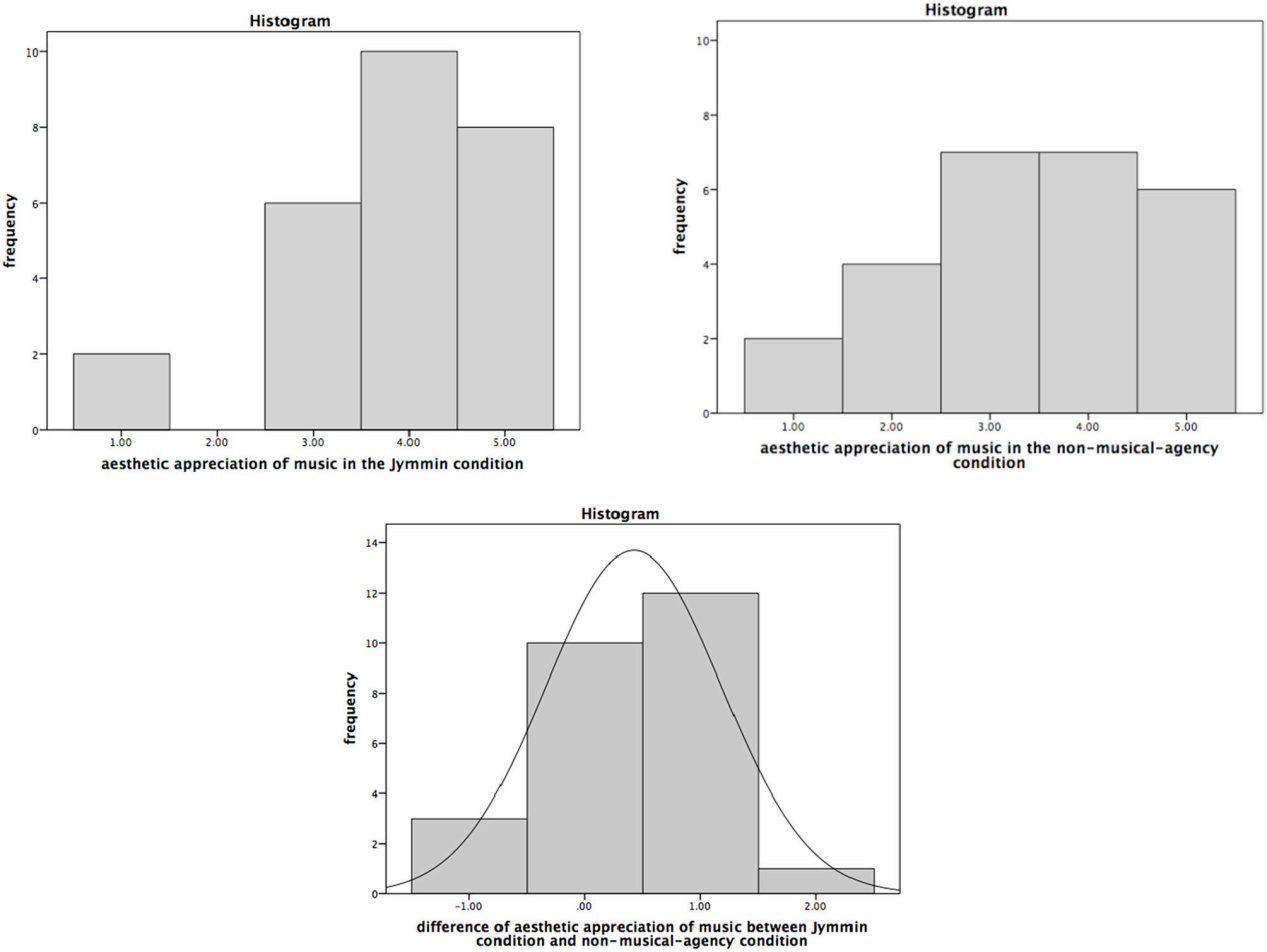
**The figure depicts scores on the esthetic appreciation of music during the ***Jymmin*** or ***non-musical-agency*** condition as measured on a 5-point Likert scale**. A Sign Test revealed a significant difference between the *Jymmin* and *non-musical-agency* condition. Patients liked the music significantly more during the *Jymmin* condition compared to the *non-musical-agency* condition.

A Sign Test showed that participants (*N* = 26) esthetically appreciated the music during the *Jymmin* condition (*Mdn* = 4.00) significantly more as compared to the music during the *non-musical-agency* condition (*Mdn* = 3.50), median of the differences = 0.50, *P* (13/26) = 0.50 (0.31, 0.69), *p* = 0.021 (two-tailed). Thirteen participants liked the musical material more in the *Jymmin* condition, 10 had a similar appreciation in both conditions, and 3 participants liked the musical material more during the *non-musical-agency* condition (see also Figure 1, see Supplementary Material for raw data). The odds ratio is therefore 4.33 (1.10, 17.02), which constitutes a large effect size.

### Experiment 2

**Figure 2:**
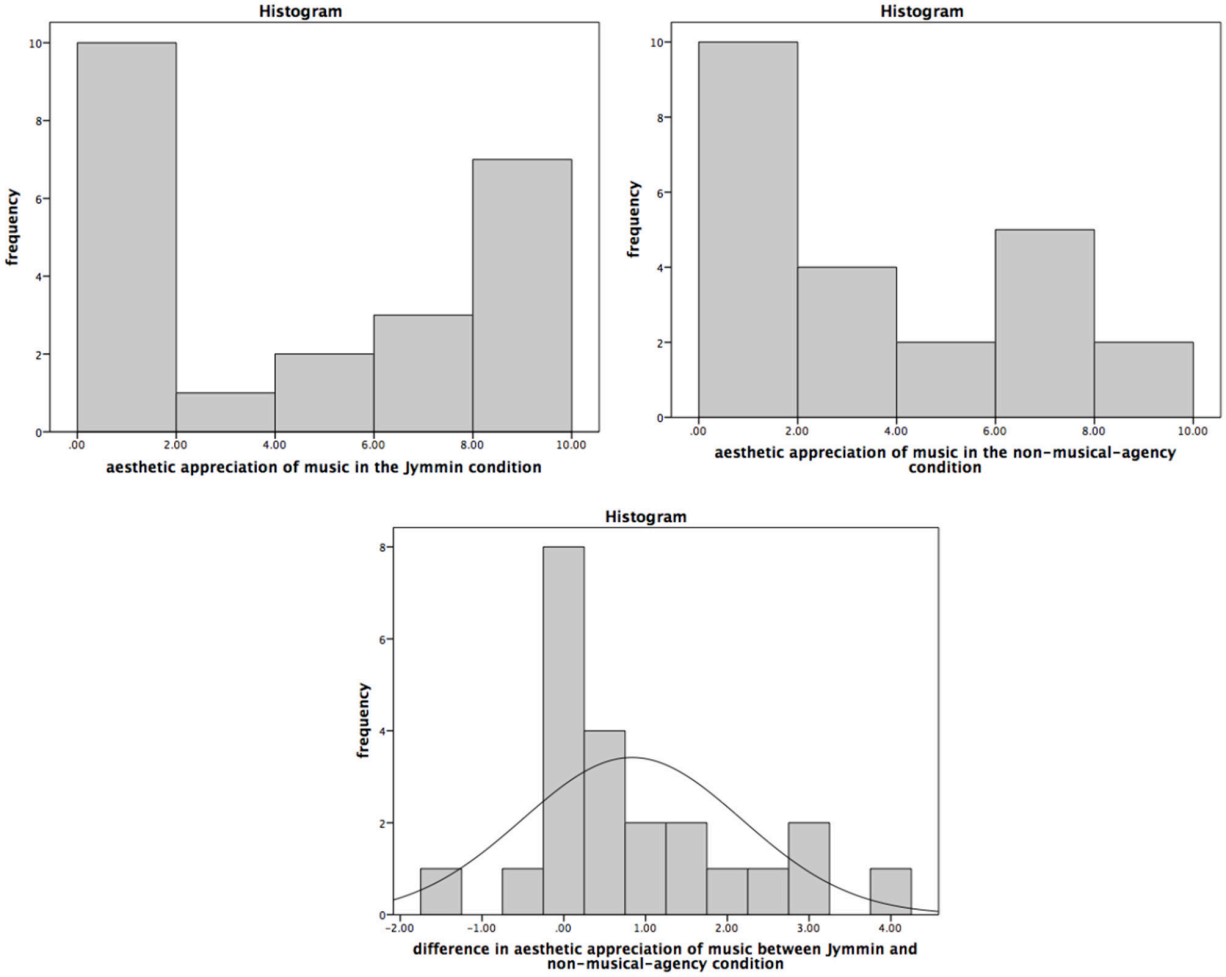
**The figure depicts scores on the esthetic appreciation of music during the ***Jymmin*** or ***non-musical-agency*** condition as measured on a ***VAS in cm*****. A Sign Test revealed a significant difference between the *Jymmin* and *non-musical-agency* condition. Patients liked the music significantly more during the *Jymmin* condition compared to the *non-musical-agency* condition.

A Sign Test revealed that chronic pain patients (*N* = 23) esthetically appreciated the music during the *Jymmin* condition (*Mdn* = 4.50) significantly more than during the *non-musical-agency* condition (*Mdn* = 2.90), median of the differences = 0.40, *P* (16/23) = 0.70, (0.51, 0.89), *p* = 0.027 (two-tailed). Sixteen participants liked the musical material more in the *Jymmin* condition, 2 had a similar appreciation in both conditions, and 5 participants liked the musical material more during the *non-musical-agency* condition (see also Figure 2, see Supplementary Material for raw data). The odds ratio is therefore 3.20 (1.00, 10.19), which constitutes a medium effect size.

## Discussion

During musical agency, when music is performed during *Jymmin*, the esthetical appreciation of the musical excerpts was greater compared to passively listening to the same kind of music without musical agency. This could be analyzed because (with the exception of the first experimental participant groups who listened to their own music performance during the passive listening condition) across all groups all music pieces were once listened to during the *Jymmin* conditions (where they were created live) and once as a recording (of the performance of another group) during workout in the passive listening conditions. In other words, when participants performed the music themselves, they were much more appreciative of the music than when they listened to a recording of the same type of music. To every hobby musician this phenomenon will probably ring a bell. Such a “Band Effect” may be observed by musicians and aspiring musicians as the subjective discrepancy between the quality of a musical performance when played live at a concert or in a rehearsal space (e.g., “it was a great concert”) and later listening to the music recording (e.g., “the recording sounds really bad”). Here we argue that this band effect may be the result of a positivity bias the *Jymmin* performers experience. Positivity bias has been described as a tendency of the individual to selectively interpret themselves and their environment in such a way that allows them to maintain a positive self-image.

### Benefits of the band effect

That the outcome of personal musical agency tends to be perceived as rewarding even if it does not comply to personal esthetic appreciation is highly beneficial to musical feedback interventions. For example, in a clinical setting the musical styles to be performed will not have to be precisely matched to individual taste. Therefore, performing such interventions in group-settings becomes much easier. Because positivity bias is reliably transferred to in-group experiences, this furthers positive social experiences that will again facilitate the examined music-sports training in groups.

From the perspective of a music composer and a music teacher *Jymmin* would also offer quite unique opportunities. Because the intervention is not limited to any musical style and material, it can also be applied to explore music that the listener is unfamiliar with and would otherwise be reluctant to listen to. This would be an interesting tool for composers to encourage participants to strongly engage with for example avant-garde music. It also constitutes an interesting tool for music educators and teachers such that it would create positive experiences in the act of acquiring new musical experiences. Note that because this procedure is largely based on practical experiences, this would also be beneficial in terms of early music experiences in children.

### Possible mechanisms underlying the band effect

Two parameters critically define the *Jymmin* intervention, musical agency, and physical exertion. While in the current study it is not possible to isolate the effect of either one alone, it is probable that physical exertion plays an important part in mediating the perceived positivity bias. A high physical workload dedicated to creating a musical piece constitutes a strong commitment on the side of the performer. Psychological research demonstrated that commitment to a cause, especially when displayed publicly (as it is the case during the social *Jymmin* interaction) increases the subjectively perceived quality of this cause (Cialdini, 2001). It is reasonable to believe that accordingly the quality of the musical pieces in terms of esthetic appeal for the performers increased after commiting a high level of physical activity to its creation.

Furthermore, it is possible that participants perceived a transfer of feeling good due to hormonally mediated “runner's high”-like sensations to the quality of the music they heard during their performance, attributing the perceived mood increase to the musical stimulation. Finally, the process of music making creates a richness of semantic associations with a musical piece personally created, for example with respect to the physical effort that was necessary to create it, the challenge to motivate each other in critical moments during the performance, mimic communication that occurred during the performance, etc. Given that it was shown that the capability to identify meaning in music can increase its appreciation (Fritz et al., 2009), this may also lead to an enhanced esthetic appreciation.

In conclusion, we present two datasets from different patient populations that demonstrate a positivity bias when assessing the esthetic quality of musical pieces performed personally with strong physical exertion compared to passively listening to similar music excerpts. Such a positivity bias due to this specific type of musical agency (*Jymmin*) implies that in these musical feedback interventions personal musical taste (such as e.g., a preference for certain musical styles) may not be crucial. This on the one hand creates the opportunity to clinically apply music-sports training in groups. On the other hand it is also an opportunity in musical education to increase the motivation to explore unfamiliar musical styles that are otherwise perceived as unpleasant.

## Author contributions

TF, LS, AV designed the experiment, TF, LS wrote the manuscript, LS, TF did the data analysis.

### Conflict of interest statement

The authors declare that the research was conducted in the absence of any commercial or financial relationships that could be construed as a potential conflict of interest. Part of the Jymmin technology applied in the experiments is subject to a patent application by the Max Planck Society.
